# Mercury in forest mushrooms and topsoil from the Yunnan highlands and the subalpine region of the Minya Konka summit in the Eastern Tibetan Plateau

**DOI:** 10.1007/s11356-016-7580-6

**Published:** 2016-09-12

**Authors:** Jerzy Falandysz, Martyna Saba, Hong-Gao Liu, Tao Li, Ji-Peng Wang, Anna Wiejak, Ji Zhang, Yuan-Zhong Wang, Dan Zhang

**Affiliations:** 1 Laboratory of Environmental Chemistry and Ecotoxicology, Gdańsk University, Gdańsk, Poland; 2 College of Agronomy and Biotechnology, Yunnan Agricultural University, Kunming, China; 3 College of Resources and Environment, Yuxi Normal University, Yuxi, China; 4 Institute of Mountain Hazards and Environment, Chinese Academy of Sciences, Chengdu, China; 5 Institute of Soil and Fertilizer, Sichuan Academy of Agricultural Sciences, Chengdu, China; 6 Institute of Medicinal Plants, Yunnan Academy of Agricultural Sciences, Kunming, China; 7 Yunnan Technical Center for Quality of Chinese Materia Medica, Kunming, Yunnan China

**Keywords:** Asia, Foraging, Heavy metals, Mushrooms, China, Yunnan

## Abstract

This study aimed to investigate and discuss the occurrence and accumulation of mercury in the fruiting bodies of wild-growing fungi (Macromycetes) collected from montane forests in two regions of southwestern China with differences in soil geochemistry, climate and geographical conditions. Fungal mycelia in soils of the subalpine region of the Minya Konka (Gongga Mountain) in Sichuan and in the highlands of Yunnan efficiently accumulated mercury in fruiting bodies (mushrooms). The examined sites in Yunnan with highly mineralized red and yellow soils showed Hg contents ranging from 0.066 to 0.28 mg kg^−1^ dry biomass (db) which is roughly similar to the results obtained for samples collected from sites with dark soils relatively rich in organic matter from a remote, the subalpine region of Minya Konka. Due to the remoteness of the subalpine section of Minya Konka, as well as its elevation and climate, airborne mercury from long-range transport could be deposited preferentially on the topsoil and the Hg levels determined in soil samples taken beneath the fruiting bodies were up to 0.48 mg kg^−1^ dry matter. In Yunnan, with polymetallic soils (Circum-Pacific Mercuriferous Belt), *Amanita* mushrooms showed mercury in caps of fruiting bodies of up to 7.3 mg kg^−1^ dry biomass. Geogenic Hg from the mercuriferous belt seems to be the overriding source of mercury accumulated in mushrooms foraged in the regions of Yunnan, while long-range atmospheric transport and subsequent deposition are the mercury sources for specimens foraged in the region of Minya Konka.

## Introduction

The anthropogenic emissions of Hg are a process that has been ongoing for decades now and has caused increases in topsoil Hg contents, thereby creating a pool of Hg with the likelihood of the formation of MeHg that accumulates in the aquatic food web (Olivero-Verbel et al. 2002, 2015). Geogenic Hg is usually low in soils worldwide due to the low Hg content of the parent bedrock material, which is, on average, <0.05 mg kg^−1^ dry matter (dm) in the top 0–10 cm layer of forest soils collected beneath mushrooms across Poland, and with an average of about 0.08 mg kg^−1^ dm in the Earth’s crust (Falandysz and Bielawski 2007; Falandysz and Brzostowski 2007; Rytuba 2003). In the regions of the Circum-Pacific Mercuriferous Belt in China, geogenic Hg from mineral deposits is usually elevated or even high in soils in the Yunnan and Guizhou Provinces in the southwestern parts of China (Fan 1991; Gustin et al. 1999; Shi et al. 2013; Wen and Chi 2007).

In the regions of Yunnan Province, the Hg content of the top (0–10 cm) layer of red and yellow soils in forested areas varied widely: from 0.096 to 0.91 mg kg^−1^ dm (Beicheng, Caoba, Daying, Eshan, Huangcaoba, Jiangchuan, Jiulongchi, Xinping and Yimen); from 0.15 to 3.4 mg kg^−1^ dm in Dayingjie in the Yuxi city; 0.25 mg kg^−1^ dm for Qilin in the Qujing city; 0.22 mg kg^−1^ dm for Dongshan in the Wenshan prefecture; 0.20 to 0.48 mg kg^−1^ dm for Pudacu in the Diqing prefecture; 1.3 mg kg^−1^ dm for Weixi in the Diqing prefecture; 0.065 to 0.91 mg kg^−1^ dm for Nanhua and Yuanmou in the Chuxiong prefecture; 0.19 to 1.1 mg kg^−1^ dm for Anning, Fumin and Shilin in the Kunming city; 0.33 to 0.35 mg kg^−1^ dm for Shiping in the Honghe prefecture; 0.13 to 2.6 mg kg^−1^ dm for Heqing, Midu and Yunlong in the Dali prefecture; 0.43 mg kg^−1^ dm for Yuanmou; from 0.083 to 1.2 mg kg^−1^ dm for Lufeng in the Chuxiong prefecture; 0.22 to 1.2 mg kg^−1^ dm for Dongshan in the Wenshan prefecture; 0.68 mg kg^−1^ dm for Yongren in the Chuxiong prefecture; 0.073 to 0.21 mg kg^−1^ dm for Malong in the Qujing prefecture; 0.31 to 2.1 mg kg^−1^ dm for Longyang region, Baoshan city; 0.13 mg kg^−1^ dm for Zhenyuan; from 0.43 to 0.53 mg kg^−1^ dm for Pu’er city; 2.4 mg kg^−1^ dm for Lanping in the Nujiang prefecture; and 0.42 to 0.98 mg kg^−1^ dm for Gejiu and Shiping in the Honghe prefecture (Falandysz et al. 2015a, b; Kojta et al. 2015).

The mycelial network is a device used by mushrooms in the absorption of chemical elements from substratum wherever the mycelium lives (soil, decaying litter, decaying wood, etc.). The mycelium could form rhizomorphs but no other mechanism (absorbing body) exists. Mushrooms differ in the substrate they grow on as well as in the depth where the mycelia live in soil. Mushrooms have no known device with which to absorb Hg compounds from the atmosphere, and any adsorption of Hg compounds or atmospheric particles containing this element at the surface (cap) of an ephemeral fruiting body would be negligible and without any practical significance for the background forested areas. For some mushrooms with small fruiting bodies, cleaning the litter and soil debris from the fruiting bodies using a plastic knife and brush may not be satisfactory/effective. Hence, effective cleaning can be achieved by washing the fruiting bodies with distilled water, as this is necessary to remove all traces of soil particles and litter debris before further processing and chemical examination.

Little is known of the significance of the mercuriferous belt areas on the occurrence of Hg and other metallic elements and metalloids in mushrooms. Little or almost nothing is known about the impact of the global mercury fallout, which is deposited and retained in the upper organic layer of soils (especially forest soils), which subsequently moves slowly down the soil horizon where it could become available to the mycelia. Suggestions that atmospheric particles could be a significant source of Hg to mushrooms (deposited on and then adsorbed through the cap) lack evidence for the mushrooms typically foraged in the forests and fields, especially considering the short lifetime (1 day to 2 weeks) of the ephemeral fruiting bodies. These issues are addressed shortly below.

At sites polluted by cinnabar mining or mine tailings, Hg could be accumulated by some mushrooms in amounts that far exceed those sequestered by the same species when growing in background areas. For example bay bolete from the genus *Imleria* (*Imleria badia* (Fr.) Vizzini, previous names *Xerocomus badius* (Fr.) E.-J. Gilbert, and *Boletus badius* (Fr.) Fr.) from a medieval gold and copper mine dump area near the town of Złotoryja in southern Poland contained Hg at 0.71 ± 0.49 mg kg^−1^ dry biomass (db) in caps and 0.41 ± 0.29 mg kg^−1^ db in stipes; the values were 1.3 ± 1.1 mg kg^−1^ db in whole fruiting bodies of *I. badia* from a site adjacent to the historical silver-mining area in South Bohemia in the Czech Republic; at 0.4 ± 0.1–0.6 ± 0.1 mg kg^−1^ db in individuals from a site polluted by flotation tailings in the southern part of Poland; and at 15–35 mg kg^−1^ db in a site of a former cinnabar mining area in Germany (Fischer et al. 1995; Kojta et al. 2012; Mleczek et al. 2015; Svoboda et al. 2006). A highly elevated concentration level of Hg occurred in mushrooms that grew in former mercury-mining areas: 110 mg kg^−1^ db in *Melanoleuca grammopodia* (Bull.) Murrill, from the Mountain Amiata in Italy; 52 ± 61 (up to 180) mg kg^−1^ db in *Suillus grevillei* (Klotzsch) Singer; and 48 ± 122 (up to 470) mg kg^−1^ db in *Lactarius quietus* from the Rudñany site in southeast Slovakia (Árvay et al. 2014; Bargagli and Baldi 1984). 

Mushrooms collected at sites polluted with Hg from long-term industrial activities (e.g. mercury and copper ore smelting industry) have been observed to contain Hg contents as high as 5.2 ± 3.5 (up to 11) mg kg^−1^ db for *I. badia* (*B. badius*), 32 ± 10 (up to 62) mg kg^−1^ db for *Boletus edulis* Bull. and 85 ± 36 (up to 110) mg kg^−1^ db for *Lepista nuda* (Bull.) Cooke (Kalac̆ et al. 1996).

Southwestern China is one of the regions of the world within the mercuriferous belt (Gustin et al. 1999). Soils in this region are highly weathered and mineralized (Fan 1991). Nevertheless, there is currently dearth of data on Hg accumulation in wild-growing mushrooms from the Circum-Pacific Mercuriferous Belt region of China. Yunnan, China has one of the largest bio-diversities of mushrooms in the world. Wild-growing mushrooms are popular and traditionally widely foraged in the regions of Asia. In Yunnan, China, fungi are an important regional organic food that needs to be better characterized regarding the composition and accumulation of trace elements and other compounds in fruiting bodies (Wang et al. 2014).

In southwestern China, some montane regions are subjected to elevated deposition rates of airborne Hg because of long-range atmospheric transportation and deposition due to specific microclimatic and geographical conditions (Falandysz et al. 2014a; Zhang et al. 2013). Airborne Hg on the forest floor is bound by humus (organic) layer constituents, which efficiently retain it for subsequent infiltration down the soil horizons (Suchara and Sucharová 2002).

Both saprobic and mycorrhizal fungi with shallow mycelia depend more or less on decaying litter and organic layer of soils as a source of mineral compounds, while those with mycelia deeper in the soil horizon can depend on organic (humus) and/or mineral fractions of the forest soil. Results from a recent study suggest that for fungi of genus *Boletus*, *Leccinum* and *Xerocomus* which are of the mycorrhizal (symbiotic) type and have mycelia deeper in soil, the geogenic Hg in the mercuriferous belt could be an overriding source of this element accumulated in fruiting bodies (Falandysz et al. 2015a, b; Kojta et al. 2015; Ostos et al. 2015).

The top organic layer of forest soils is often enriched with Hg largely because of aerial deposition from anthropogenic sources over time. For example the Hg content of the top (0–1 cm) organic layer of the red and yellow lateritic soil collected from a forest within the outskirts of Yuxi in Yunnan was 0.16 mg kg^−1^ dm, while the Hg content of the bedrock (at depth of 2 to 3 m) below this soil varied from 0.0078 to 0.020 mg kg^−1^ dm (JF, unpublished). The dark montane forest soils of the subalpine region of Minya Konka in the Eastern Tibetan Plateau is of a different type and bedrock when compared to the red and yellow lateritic soils that dominate the central and southern part of Yunnan. However, both regions differ substantially in their elevation above sea level as well as in their climatic conditions; therefore, their susceptibility to long-range atmospheric pollution could differ.

The aim of this study was to fill the knowledge gap and provide some basic information on the occurrence, accumulation and distribution of Hg in fruiting bodies of several species of fungi collected from the Yunnan highlands which is subjected to direct impact from the Circum-Pacific Mercuriferous Belt and the subalpine region of the Minya Konka summit. The latter locations are within Sichuan Province and are considered pristine and outside the mercuriferous belt.

## Materials and methods

### Mushrooms and topsoil samples

**Fig. 1:**
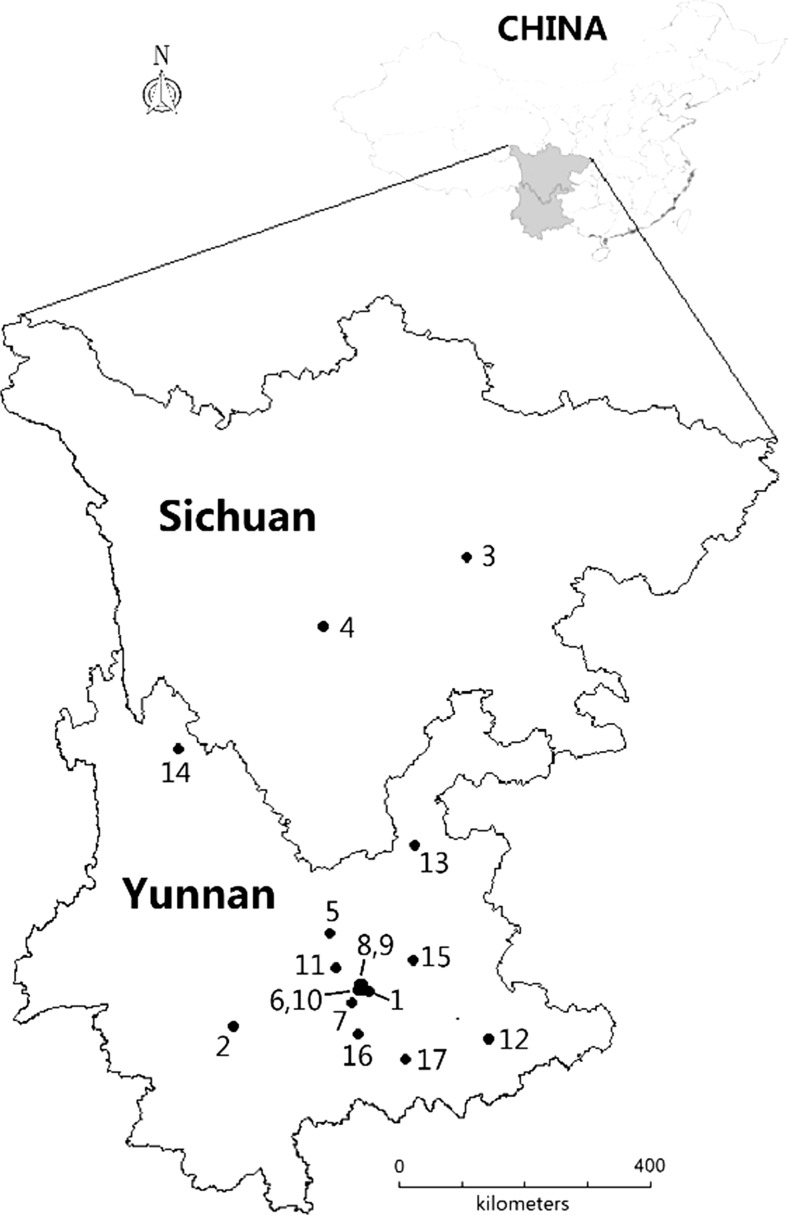
The sampling sites of mushrooms in the Yunnan and Minya Konka in the Sichuan Province of China (*1* Jiuxi, *2* Zhenyuan, Pu’er, *3* Chengdu, *4* Minya Konka at 3480 m a.s.l., *5* Lufeng, Chuxiong, *6* Dayingjie, *7* Eshan, Yuxi, *8* Jiulongchi, Yuxi, *9* Beicheng, Yuxi, *10* Hongta region, Yuxi, *11* Yimen, Yuxi, *12* Yanshan, WenShan, *13* Huize, Qujing, *14* Pudacuo, Diqing, *15* Shilin, Kunming, *16* Shiping, Honghe, *17* Gejiu, Honghe)—*numbers* inside are explained in Table 1

Twenty three species of edible mushrooms were collected from spatially distributed locations across Yunnan Province, China during the collection seasons (June–September) of a period ranging from 2011 to 2014. Another six species were collected from the Minya Konka summit at an elevation of from 2800 to 3480 m above sea level in 2012 (Fig. 1). Macromycetes develop fruiting bodies seasonally (largely in late summer and early autumn which is the best for reproduction) but not necessarily annually. A pack of dried fruiting bodies of cultivated *Auricularia polytricha* (Mont.) Sacc. (current name *Auricularia nigricans* (Sw.) Birkebak, Looney & Sánchez-García) was also bought from a grocery store in Chengdu in Sichuan in 2012, and this was divided into 15 parts and then examined individually. Alongside the mushrooms from these locations, 21 soil samples (the upper 0–10 cm layer) beneath the fruiting bodies were also collected. No specific permits were required for sampling within these described areas. Similarly, no endangered or protected species were sampled, and these localities where were not protected in any way.

Previous studies have shown that Hg content of fruiting bodies of fungi such as *B. edulis*, *Macrolepiota procera* (Scop.) Singer, *P. involutus* collected successively from the same areas over a period of between 2 and 4 years fluctuated but without any consistent pattern. This could therefore be considered as a natural phenomenon related to biological condition and weather (Brzostowski et al. 2011; Gucia et al. 2012; Zhang et al. 2010). Hence, a similar or related pattern could be expected in case of the fungi collected in this study.

The scientific names for species collected from Yunnan are as follows: *Amanita echinocephala* (Vittad.) Quél, *Amanita manginiana* Hariot et Patouillard, *Amanita vaginata* (Bull.:Fr) Vitt, *Clitocybe xanthophylla* Bres, *Heimioporus retisporus* (Pat. & C.F. Baker) E. Horak, *Laccaria amethystina* (Scop.) Cooke, *Laccaria laccata* (Scop.:Fr) Berk.et Br., *Laccaria proxima* (Boud.) Pat., *Laccaria vinaceoavellanea* Hongo, *Lactarius deliciosus* (L.:Fr.) Gray, *Rubinoboletus balloui* (Peck) Heinem. & Rammeloo, *Scleroderma citrinum* Pers., *Scleroderma flavidum* Ellis & Everh., *Suillus collinitus* Fr., *Suillus pictus* (Peck) A.H. Smith et Thiers, *Tapinella panuoides* (Batsch) E.-J. Gilbert, *Tricholoma matsutake* (S. Ito et Imai) Sing., *Tylopilus felleus* (Bull.) P. Karst., *Tylopinus nigerrimus* (Heim) Hongo & Endo, *Tylopilus plumbeoviolaceoides* T.H. Li, B. Song & Y.H. Shen, *Tylopilus plumbeoviolaceus* (Snell.) Sing, *Tylopilus roseolus* (Chiu) Tai, and from the Minya Konka area: *Cortinarius bovinus* Fr., *Cortinarius collinitus* Fr., *Hygrocybe conica* (Schaeff.) P. Kumm., *Leccinum scabrum* (Bull.) Gray, *Russula grisea* Fr., *Russula puellaris* Fr. and *Tricholoma pessundatum* (S. Ito et Imai) Sing. (Table 1) (Index Fungorum 2015).

**Table 1:** Mercury content in dried mushrooms and soil substratum from Yunnan and Minya Konka in China, quotient of Hg content in cap to stipe (*Q*_C/S_), and quotient of Hg content in cap/stipe to soil beneath the fruiting bodies (BCF)

Species, place and year of collection	Number	Hg (mg kg^−1^)			*Q*_C/S_	BCF	
		Whole fruiting bodies		Soils		Whole fruiting bodies	
		Caps	Stipes			Caps	Stipes
*Amanita echinocephala* (Vittad.) Quél.							
[1]^a^ Jiuxi, Yuxi; 2014	(3)^b^	5.0	1.8	WD	2.8	WD	WD
*Amanita manginiana* Hariot & Patouillard							
[2] Zhenyuan, Pu’er; 2014	(5)	7.3	4.2	WD	1.7	WD	WD
*Amanita vaginata* (Bull.:Fr) Vitt							
[1] Jiuxi, Yuxi; 2014	(3)	2.9	1.8	WD	1.6	WD	WD
*Auricularia nigricans*							
[3] Chengdu, shop; 2012	15^c^	0.033 ± 0.014 0.028 (0.016–0.068)		NA	NA	NA	NA
*Clitocybe xanthophylla* Bres							
[2] Zhenyuan, Pu’er; 2014	(20)	2.5	1.5	0.097	1.7	26	16
*Cortinarius bovinus* Fr.							
[4] Minya Konka at 3480 m a.s.l.; 2012	(> 10)	0.14		0.42	NA	0.33	
[4] Minya Konka at 3480 m a.s.l.; 2012	(> 10)	0.11		0.18	NA	0.61	
*Cortinarius collinus* Fr.							
[4] Minya Konka at 3000 m a.s.l.; 2012	(> 5)	0.045	0.53	0.015	0.085	3.0	35
[4] Minya Konka at 3000 m a.s.l.; 2012	(> 5)	0.074	0.26	WD	0.28	WD	WD
*Heimioporus retisporus* (Pat. & C.F. Baker) E. Horak							
[5] Lufeng, Chuxiong; 2013	(22)	0.73	0.34	0.13	2.1	5.6	2.6
*Hygrocybe conica *(Schaeff.) P. Kumm							
[4] Minya Konka at 2800 m a.s.l.; 2012	(9)	0.75		WD	NA	WD	
[4] Minya Konka at 2800 m a.s.l.; 2012	(10)	1.7		WD	NA	WD	
[4] Minya Konka at 3000 m a.s.l.; 2012	(10)	1.6		0.28	NA	5.7	
[4] Minya Konka at 3050 m a.s.l.; 2012	(17)	1.6		WD	NA	WD	
*Laccaria amethystine* (Scop.) Cooke							
[6] Dayingjie, Yuxi; 2014	(55)	0.10	0.056	WD	1.8	WD	WD
[2] Zhenyuan, Pu’er; 2014	(170)	0.034	0.032	WD	1.1	WD	WD
*Laccaria laccata* (Scop.:Fr) Berk. & Br.							
[7] Eshan, Yuxi; 2014	(176)	0.18	0.082	WD	2.2	WD	WD
[8] Jiulongchi, Yuxi; 2014	(380)	0.12	0.068	WD	1.8	WD	WD
[6] Dayingjie, Yuxi; 2014	(201)	0.15	0.060	WD	2.5	WD	WD
*Laccaria proxima* (Boud.) Pat.							
[1] Jiuxi, Yuxi; 2014	(80)	0.11	0.086	0.043	1.3	2.6	2.0
*Laccaria vinaceoavellanea* Hongo							
[9] Beicheng, Yuxi; 2014	(84)	0.13	0.050	WD	2.6	WD	WD
[2] Zhenyuan, Pu’er; 2014	(170)	0.034	0.032	0.12	1.1	0.28	0.27
*Lactarius deliciosus *(L.:Fr.) Gray							
[2] Zhenyuan, Puer; 2014	(35)	0.83	0.78	WD	1.1	WD	WD
*Leccinum scabrum* (Bull.) Gray							
[4] Minya Konka at 3000 m a.s.l.; 2012	(2)	0.22	0.12	0.15	1.8	1.5	0.80
[4] Minya Konka at 3000 m a.s.l.; 2012	(6)	0.37	0.29	0.33	1.3	1.1	0.88
[4] Minya Konka at 2900–3000 m a.s.l.; 2012	(4)	0.13	0.37	0.13	0.35	1.0	2.8
*Rubinoboletus balloui* (Peck) Heinem. & Rammeloo							
[1] Jiuxi, Yuxi; 2014	(17)	3.8	2.0	WD	1.9	WD	WD
*Russula grisea* Fr.							
[4] Minya Konka at 3000 m a.s.l.; 2012	(>10)	0.15	0.088	0.23	1.7	0.65	0.38
*Russula puellaris* Fr.							
[4] Minya Konka at 3000 m a.s.l.; 2012	(11)	0.077	0.12	0.37	0.64	0.21	0.32
*Scleroderma citrinum* Pers.							
[7] Eshan, Yuxi; 2014	(25)	0.090		WD	NA	WD	
*Scleroderma flavidum* Ell. & Ev.							
[1] Jiuxi, Yuxi; 2014	(9)	0.078		WD	NA	WD	
[10] Hongta region, Yuxi; 2014	(15)	0.19		WD	NA	WD	
[6] Dayingjie, Yuxi; 2014	(18)	0.047		WD	NA	WD	
*Suillus collinitus* Fr.							
[1] Jiuxi, Yuxi; 2013	(11)	0.089	0.040	0.045	2.2	2.0	0.90
[1] Jiuxi, Yuxi; 2014	(41)	0.32	0.20	0.11	1.6	2.9	1.8
*Suillus pictus* (Peck) A.H. Smith & Thiers							
[2]Zhenyuan, Pu’er; 2014	(16)	0.42	0.14	WD	3.0	WD	WD
[1] Jiuxi, Yuxi; 2014	(19)	0.22	0.10	WD	2.2	WD	WD
*Tapinella panuoides* (Batsch) E.-J. Gilbert							
[2] Zhenyuan, Pu’er; 2014	(23)	0.085		WD	NA	WD	
*Tricholoma matsutake* (S.Ito & Imai) Sing.							
[11] Yimen, Yuxi; 2012	(10)	0.73	0.44	WD	1.7	WD	WD
[12] Yanshan, WenShan; 2012	(10)	1.0	0.62	0.27	1.6	3.7	2.3
*Tricholoma pessundatum* (S. Ito & Imai) Sing.							
[4] Mt. Gongga at 2800 m a.s.l.; 2012	(24)	1.2	0.61	0.17	2.0	7.0	3.6
*Tylopilus felleus* (Bull.:Fr.) Karst							
[10] Hongta region, Yuxi; 2012	(10)	0.29	0.26	0.066	1.1	4.4	3.9
[13] Huize, Qujing; 2013	(5)	2.6	2.7	WD	0.96	WD	WD
[13] Huize, Qujing; 2013	(10)	2.2	1.5	WD	1.5	WD	WD
*Tylopinus nigerrimus* (Heim) Hongo & Endo							
[14] Pudacuo, Diqing; 2012	(10)	2.8	1.4	0.28	2.0	10	5.0
*Tylopilus plumbeoviolaceoides* T.H. Li, B. Song & Y.H. Shen							
[15] Shilin, Kunming; 2012	(10)	0.68	0.49	0.15	1.4	4.5	3.3
[13] Huize, Qujing; 2013	(10)	1.3	1.0	WD	1.3	WD	WD
*Tylopilus plumbeoviolaceus* (Snell.) Sing							
[15] Shilin, Kunming; 2012	(10)	0.68	0.49	0.15	1.4	4.5	3.3
*Tylopilus roseolus* (Chiu) Tai.							
[16] Shiping, Honghe; 2012	(10)	0.61	0.42	WD	1.4	WD	WD
[17] Gejiu, Honghe; 2012	(10)	0.41	0.31	WD	1.3	WD	WD

Mean value ± SD, median value, and range

*BCF* bioconcentration factor, *WD* without data, *NA* not applicable

^a^In bracket, a number of the sampling site is given (see Fig. 1)

^b^Number of individuals in a composite sample

^c^For *Auricularia nigricans*, 15 pooled samples have been examined (total quantity of mushrooms was 0.5 kg)

Fruiting bodies that were in good condition were collected and then cleaned carefully from any visible plant vegetation and soil debris with a plastic knife. To get insight into the distribution of total mercury (THg) between the two major morphological parts of the fruiting bodies of mushrooms (for some of the species), specimens were separated into cap (with skin) and stipe and further pooled accordingly with the aim of obtaining representative composite samples representing each species, sampling location and time of collection (Table 1). Thereafter, the mushroom samples were placed into electrically heated plastic trays used commercially in drying vegetables and then dried at 65 °C (Yunnan) or into a paper bag and dried in electrically heated laboratory driers at 65 °C (Minya Konka) to constant mass. Drying of mushrooms at 65 °C to constant weight is a standard procedure, e.g. for production of official certified fungal reference materials used in AC/AQ protocols in this study. Some authors dry mushrooms at 40 °C, while drying at 105 °C is not recommended because some organic compounds evaporate alongside water. Dried fungal materials were pulverized in a porcelain mortar and kept in sealed polyethylene bags under dry conditions. The soil samples, free of any visible organisms, small stones, sticks and leaves, were air dried at room temperature for several weeks under clean (dust free) conditions in the laboratory. Next, the soil samples were ground in a porcelain mortar, sieved through a 2-mm plastic sieve and kept in sealed polyethylene bags under dry conditions.

### Mercury determinations

All the reagents used in this study were of analytical reagent grade, unless otherwise stated. Double distilled water was used for the preparation of the solutions. Mercury standard solution of 1.0 mg Hg mL^−1^ was obtained from a 10 mg Hg mL^−1^ standard stock solution. Blank and 100, 150 and 200 μL of 1.0 mg mL^−1^ Hg standard solutions were injected into the analyser for the construction of a calibration curve, which was prepared fresh each week.

The determinations of total Hg content of fungal and soils samples were performed using cold-vapour atomic absorption spectroscopy (CV-AAS) by direct sample thermal decomposition coupled with gold wool trap of Hg and its further desorption and quantitative measurement at wavelength of 296 nm. The analytical instrument used was mercury analyser (MA-2000, Nippon Instruments Corporation, Takatsuki, Japan) equipped with auto sampler and operated at low mode (Brzostowski et al. 2011; Nnorom et al. 2013).

A running quality assurance/quality control (QA/QC) was performed through the analysis of blank samples and certified reference materials such as CS-M-4 (dried fruit bodies of King Bolete *B. edulis* mushroom) and CS-M-1 (dried fruit bodies of Cow Bolete *Suillus bovinus* mushroom) produced by the Institute of Nuclear Chemistry and Technology, Warsaw, Poland. The declared total Hg content of material CS-M-4 is 2.849 ± 0.104 mg kg^−1^ db, while present determinations (*n* = 5) showed 2.833 ± 0.023 mg kg^−1^ db. In the case of the material CS-M-1 the declared content is 0.164 ± 0.004 mg Hg kg^−1^ db, while present determination (*n* = 13) showed 0.185 ± 0.011 mg kg^−1^ db. The limit of detection (LOD) of this study was 0.0015 mg Hg kg^−1^ db, and the quantification limit (LOQ) was 0.005 mg Hg kg^−1^ db. One blank sample and one certified reference material sample were examined with each set of 3–5 samples studied. To estimate the potential of the fruiting bodies of macromycetes to sequester Hg or any other chemical element, it is common to calculate the bio-concentration factor (BCF).

## Results and discussion

### Mercury in soil substrata

The Hg content of the 0–10 cm layer (no litter) of soil samples from stands in Minya Konka for most of the samples ranged from 0.13 to 0.42 mg kg^−1^ dry matter (dm) and from a stand of *C. collinus* was an order of magnitude lower, i.e. at 0.015 mg kg^−1^ dm (Table 1). The examined sites in Yunnan with highly mineralized red and yellow soils showed Hg contents ranging from 0.066 to 0.28 mg kg^−1^ db which is roughly similar to the results obtained for samples collected from sites with dark soils relatively rich in organic matter from a remote, subalpine region of Minya Konka.

### Mercury in fruiting bodies of mushrooms

Seven mushrooms species collected from the subalpine region in Minya Konka belonged to 5 genera and the other 22 species from Yunnan were from 11 genera. In all cases, the amounts/quantities of each sample, whether whole fruiting bodies or separated caps and stipes, were relatively large and sufficient for chemical analyses. For several species of mushrooms, it was possible to collect fruiting bodies from more than one site/location.

Mushrooms such as *C. bovinus*, *C. collinus*, *L. scabrum*, *R. grisea* and *R. puellaris* from Minya Konka showed an order of magnitude of lower contamination levels of Hg (<1.0 mg kg^−1^ db) than *H. conica* which showed 1.6 mg kg^−1^ db and *T. pessundatum* with 1.2 mg Hg kg^−1^ db in caps and 0.61 mg Hg kg^−1^ db in stipes (Table 1). The pattern of Hg distribution between cap and stipe in fruiting bodies of *C. collinus* was different from that usually observed in mushrooms. The stipes of *C. collinus* showed Hg at an order of magnitude greater concentration than the caps (value of the *Q*_C/S_ index between 0.085 and 0.28; *Q*_C/S_ is a value of cap to stipe Hg concentration quotient).

Mushroom species collected from Yunnan with mycelia living deeper in the soil (including species such as *Amanita* spp., *C. xanthophylla*, *R. balloui*, *Tricholoma* spp. and *Tylopilus* spp.) showed higher contamination with Hg compared to species having shallow mycelia (such as *Laccaria* spp. and *Scleroderma* spp). Mushrooms collected from Yunnan on average contained higher Hg values in the fruiting bodies compared to samples from Minya Konka. Species such as *A. echinocephala*, *A. manginiana* and *A. vaginata* with Hg levels in caps of 5.0, 7.3 and 2.9 mg kg^−1^ db and Hg in stipes of 1.8, 4.2 and 1.8 mg kg^−1^ db respectively, need to be considered as substantially contaminated.

The amounts of Hg observed in two sets of *T. felleus* collected from Huize (in the Qujing region) are orders of magnitude that are higher than the values observed in individual samples from the Hongta region (in Yuxi), which indicates that the elevated geogenic Hg content of the soil substratum could be the source/reason. Similarly, *T. plumbeoviolaceoides* from Huize (in Qujing) showed higher Hg contamination compared to samples from the distant site of Shilin in the region of Kunming (Table 1).

The *Laccaria* spp. are mycorrhizal mushrooms with shallow mycelia and they feed largely on decomposing litter. All four *Laccaria* spp. in this study showed low contamination with Hg. Nevertheless, the Hg content of individual samples of *L. amethystina* and *L. vinaceoavellanea* collected from montane sites in the rural Zhenyuan region in Pu’er were 0.034 and 0.034 mg Hg kg^−1^ db in caps and 0.032 and 0.032 mg Hg kg^−1^ db in stipes which indicate less contamination compared to samples from other sites within the urbanized and industrial areas of Yuxi. *S. citrinum* (with Hg content of 0.090 mg Hg kg^−1^ db) and *S. flavidum* (with Hg range from 0.047 to 0.078 mg kg^−1^ db) can be considered to be less contaminated with Hg compared to other mushrooms from Yunnan Province.

Fruiting bodies of cultivated Wood Ear fungus *A. nigricans* (a very popular mushroom in China readily eaten raw or fried) bought from a shop in Chengdu in Sichuan were less contaminated with mercury (median value of 0.028 mg kg^−1^ db) (Table 1).

### Bio-concentration of Hg by mushrooms in fruiting bodies

The BCFs values for Hg were < 1 for mushrooms from Minya Konka (such as *C. bovinus*, *R. grisea* and *R. puellaris*); about ∼1 for *L. scabrum*; > 1 for other species and up to 35 for *C. collinus* (stipes). The BCF values for most mushrooms from Yunnan, for which there were available data on Hg in soil, showed values > 1 and up to 26 (for caps) and 16 (for stipes) for *C. xanthophylla.* An exception was observed for the *Laccaria* spp.: the BCF for *L. vinaceoavellanea* was < 1 while the BCF for *L. proxima* was ∼2. Most of the mushrooms in this study with available data on Hg in their soil substrate bio-included Hg.

### Mercury in forest soils

The forest soils sampled in this study contained Hg in the top 0–10 cm layer at a relatively narrow range with elevated levels of up to 0.42 mg kg^−1^ dm observed for Minya Konka and up to 0.28 mg kg^−1^ dm for Yunnan. The topsoil sampled in the subalpine region of Minya Konka with one exception contained Hg at concentrations above 0.13 mg kg^−1^ dm, which is considered a critical level because of the likely harmful effects on soil organisms (Tipping et al. 2010).

The eastern slope of Minya Konka at the subalpine part is characterized by natural forests starting from an elevation of 2000 to 3600 m above sea level (a.s.l.). In this region, between 2000 and 2400 m a.s.l., the dominant tree species are *Lithocarpus cleistocarpus* (Seemen) Rehder & E.H. Wilson and *Acer flabellatum* J. D. Hooker & Thomson ex Hiern; from 2400 to 2900 m a.s.l. are the broad-leaf and dark coniferous mixed forests and the dominant trees are *Abies fabri* (Mast.) Craib and *A. flabellatum*; and from 2900 to 3650 m a.s.l., in the subalpine dark coniferous forest, the dominant tree species is *A. fabri*. The Minya Konka site differed from the sites sampled in Yunnan because of not only the type of forest but also the type of soil, parent rock material and soil geochemical background, as well as climate which is colder at this location with greater humidity and wet precipitation (Wu et al. 2013). The annual mean precipitation at an altitude of 3000 m a.s.l. is 1949 mm and this increases to between 3500 and 4200 mm as the altitude increases up to 3500 m a.s.l. (Wu et al. 2013). The soil organic matter content (%) in Minya Konka at the altitudes above sea level of 2361, 2777, 3317 and 3775 m are 7.06, 5.54, 5.95 and 5.04 respectively (Sun et al. 2013). As mentioned earlier, the soils in Yunnan and the neighbouring provinces, especially the eastern cities are latosols and lateritic red earths and red and yellow soils, are weathered and mineralized (polymetallic) with elevated contents of geogenic Hg and Sb in the region of the Circum-Pacific Mercuriferous Belt (Fan 1991; He et al. 2004; Qiu et al. 2012; Wen and Chi 2007).

In the Minya Konka region in the Eastern Tibetan Plateau and elsewhere in the Tibetan Plateau, depositions of airborne Hg are considered to be the primary mechanism of Hg contamination of the ground, including litter and forest soils as well as some mushrooms (Falandysz et al. 2014a; Fu et al. 2008; Zhang et al. 2008). The anthropogenic, airborne Hg is an important fraction of Hg accumulated in montane soils in southwestern China and atmospheric input of anthropogenic Hg increases with altitude above sea level as evidenced for Mt. Leigong (Zhang et al. 2013). Hence, an elevated content of Hg observed in topsoil in forests on the eastern slope of Minya Konka, which is often dark and rich in organic matter and without known sources of geogenic Hg, could be a result of enhanced atmospheric depositions which are subsequently accumulated by mushrooms into their fruiting bodies. Nevertheless, information of vertical deposition of Hg in soil horizons for forests of Minya Konka is not available. However, it is known that the ability of mushroom mycelia to take up Hg and other mineral constituents and sequester them in the fruiting bodies is, apart from genetic factors, highly related to the mushroom’s life style (feeding behaviour) and depth of mycelia in the soil horizon.

The Hg content of a limited number of the samples of red and yellow soils in Yunnan examined in this study (Table 1) was above 0.08 mg kg^−1^ dm, which, as has been cited earlier, is considered typical for the Earth’s crust. The red and yellow soils beneath the fruiting bodies of many mushrooms of the genera *Boletus* and *Leccinum* from the forests of Yunnan showed Hg in the top 0–10 cm layer of up to 3.4 mg kg^−1^ dm, which is higher than the results of this study (Table 1) (Falandysz et al. 2015a, b).

### Mercury in mushrooms

There are no previous data on Hg contamination of the fruiting bodies of most of the mushroom species/genera studied that were from Yunnan or Minya Konka. Species from the genus *Amanita* are well available in Yunnan and worldwide, and some are edible (Falandysz and Drewnowska 2015a; Mao 2009). Both *A. echinocephala* and *A. manginiana* with 5.0 and 7.3 mg Hg kg^−1^ db in caps respectively, indicated a substantial Hg uptake but there are no other published data available for these two species for comparison. In *A. vaginata*, the median value for caps was 2.9 mg Hg kg^−1^ db, which is an order or two higher than values observed for several sample sets collected from distantly distributed sites in Poland (with medians values ranging from 0.079 to 0.45 mg kg^−1^ db), and 0.75 mg kg^−1^ db for a sample set from the outskirts of Umeå in Sweden (Drewnowska et al. 2014; Falandysz et al. 2001a). *Amanita fulva* Fr., from the background areas in Poland, was much less contaminated with Hg (median mercury in caps ranging from 0.13 to 0.67 mg kg^−1^ db).

*Amanita muscaria* is a toadstool that has psychedelic effects on humans and was popular among the shamans in some locations in ancient Europe and Euro-Asia and maybe until recently in some parts of Siberia (Michelot and Melendez-Howell 2003). Apart from the organic constituents with psychedelic action, the species is known as a specific accumulator of vanadium (V) and zirconium (Zr) (Cenci et al. 2009). The reported Hg content of *A. muscaria* from the background areas in Central Europe was 0.68 mg kg^−1^ db, while values ranging from 0.19 to 1.2 mg kg^−1^ dm (and up to 1.8 mg kg^−1^ dm) were reported for individual samples collected from within the World War II battle area and from sites close to military (shooting) area and War World II battle grounds in the region of the Niedżwiednik near to the city of Gdańsk (Drewnowska et al. 2013; Falandysz et al. 2003a). A Hg content in caps of 0.39 mg kg^−1^ db, (on the average) was reported for *A. muscaria* collected from the suburbs of the city of Umeå, while other locations within northern Sweden showed substantially smaller concentrations than those observed for *Amanita* spp. collected from the mercuriferous belt in this study (Falandysz et al. 2001a).

*Amanita rubescens* Pers. showed a similar capacity to accumulate Hg as *A. muscaria* and the reported values of Hg accumulated in caps of this species collected from background areas varied from 0.22 ± 0.6 (0.11–0.34) to 1.0 ± 0.6 (0.67–1.9) and 0.83 ± 1.00 (0.26–3.6) mg kg^−1^ db (Falandysz et al. 2003b). Also, *Amanita phalloides* (Vaill. ex Fr.) Link with 1.2 ± 0.4 (0.62–1.8) mg Hg kg^−1^ db seems to have a similar capacity for sequestration of Hg as mentioned above for *A. muscaria* and *A. rubescens* (Drewnowska et al. 2012b; Falandysz et al. 2003a).

Mushrooms of the genus *Cortinarius* collected from Minya Konka showed weak contamination with Hg and possible explanations could be a weak ability to accumulate Hg and the low Hg contents of the soil substratum of *C. collinus*. Meanwhile, the Hg levels in the soil where the mycelium grows were not reported. In a study by Seeger and Nützel (1976), seven species of mushroom genus *Cortinarius* showed Hg contents ranging from 0.16 to 2.3 mg kg^−1^ db, while the Hg content of one samples of *Cortinarius elatior* Fr., was 16 mg kg^−1^ db (Seeger and Nützel 1976). In a recent study, a large collection of *C. caperatus* showed wide differences in Hg content of caps depending on sampling locations and this ranged from 0.91 ± 0.62 to 2.5 ± 0.7 mg kg^−1^ db (median values ranged from 0.81 to 2.4 mg kg^−1^ db; Falandysz 2016). The observed differences in Hg contents of *Cortinarius* mushrooms as reported by other authors compared to this study can be explained by a species-specific ability to accumulate Hg, which seems to differ for various species of this genus on one side and on the abundance of geogenic or anthropogenic Hg from soil to mycelia on the other side.

The mushroom *H. conica* from Minya Konka showed elevated Hg contents and this can be attributed to elevated Hg content of soils beneath the fruiting bodies, which was dark in colour and rich in organic matter.

There is no previous data published on Hg contents of *Laccaria* spp. from Yunnan or elsewhere in China. The species *L. laccata* (whole fruiting bodies) sampled in Hungary, Germany and Turkey in Europe showed Hg contents ranging from 0.059 ± 0.030–0.067 ± 0.01, 0.13 (0.08–0.25) and 0.12 ± 0.01 mg kg^−1^ db respectively, which are within the range of values observed in this study for China and may indicate similar rates of airborne Hg deposition and contamination of the uppermost organic layer of soils (Seeger and Nützel 1976; Tüzen and Soylak 2005; Vetter and Berta 1997).

For *L. amethystina* Cooke, from regions uncontaminated with Hg in Europe, the values reported ranged from 0.042 ± 0.003 and 0.078 ± 0.008 mg Hg kg^−1^ db for two sites in Hungary, 0.10 (0.08–0.11) mg kg^−1^ db (*n =* 6) for a site in Germany and 0.19 ± 0.08 (0.11–0.27 mg kg^−1^ db) for a site in Poland (Falandysz et al. 2001b; Seeger and Nützel 1976; Vetter and Berta 1997). The Hg content in *L. deliciosus* from Pu’er in Yunnan was similar to the values reported for this mushroom in Europe: Germany (0.35 mg kg^−1^ dm), Hungary (0.45–0.61 mg kg^−1^ db), Poland (0.94 ± 0.13 mg kg^−1^ db) and Spain (0.51–0.77 mg kg^−1^ db) (Melgar et al. 2009; Mleczek et al. 2013; Seeger and Nützel 1976; Vetter and Berta 1997).

As can be observed from data reported by Falandysz et al. (2015a, b) in some regions of the Pu’er Prefecture, the geogenic Hg is lower than in many other regions of Yunnan Province and this can explain the relatively low observed contents of Hg in *L. deliciosus* from Pu’er in this study. Nevertheless, locally, the soils and their parent rock polymetallic background material are diverse both in the mountainous region of Pu’er and other parts of Yunnan and more detailed sampling would be required to gain a better insight of the accumulation of Hg and its relationships with the soil background for mushrooms in Yunnan.

The mushroom *L. scabrum* from the sites in Minya Konka showed moderate contamination with Hg and some potential for accumulation of this element. The observed Hg levels were comparable and often times lower than data for these species reported in literature: at 0.70 ± 0.27 (0.14–1.4) mg kg^−1^ db and from 0.38 ± 0.24 to 1.2 ± 0.4 mg kg^−1^ db in Poland; at 0.40 ± 0.31–0.57 ± 0.23 mg kg^−1^ db in Spain and at 0.18 ± 0.13 (0.0033–0.70) mg kg^−1^ db in Sweden (Melgar et al. 2009; Falandysz and Bielawski 2007; Falandysz et al. 2001a, 2007).

The *Scleroderma* spp. sampled in Yunnan were low in Hg when compared with several other species in this study. In an earlier study, *S. citrinum* sampled in Poland bio-excluded Hg and its carpophores showed very low contamination with Hg, with a mean of 0.0056 ± 0.0021 (0.0026–0.0094) mg kg^−1^ db (Falandysz and Chwir 1997).

Among the mushrooms of the genus *Suillus* in this study, both *S. collinitus* and *S. pictus* from Yunnan have no previous data on the content and bio-concentration potential of Hg in fruiting bodies. The species *S. collinitus* showed some potential to bio-concentrate Hg, but it seems to be less efficient at this when compared to other species of the genus *Suillus* from other studies. For example the species *S. bovinus* (L.) Roussel, *S. granulatus* (L.) Roussel, *S. grevillei*, *S. luteus* (L.) Roussel and *S. variegatus* (Sw.) Richon & Roze, for which sufficiently data are available, both showed BCF values for Hg at around 10 (caps) (Chudzyński et al. 2009, 2011; Saba et al. 2016a, b, c).

For mushrooms of the genera *Russula*, *Tapionella*, *Tricholoma* and *Tylopilus* respectively, from Yunnan or Minya Konka, there are no available previous data on accumulation and distribution of Hg in the fruiting bodies. Both *T. matsutake* from Yunnan and *T. pessundatum* from Minya Konka showed some ability to bio-concentrate Hg (BCF > 1) and the efficiency was similar to what was observed for *Tricholoma flavovirens* (Pers.) S. Lundel (current name *Tricholoma equestre* (L.) P. Kumm.) from sites in Europe with Hg in the soil substratum of 0.059 ± 0.028 mg kg^−1^ db (Maćkiewicz and Falandysz 2012). The species *T. flavovirens* from the European locations was very efficient in the absorption of Hg from low contaminated soils, i.e. soil Hg of 0.019 ± 0.003 to 0.046 ± 0.007 mg Hg kg^−1^ db and with a BCF for caps as high as 22 ± 9 to 75 ± 13 (overall range 9.0–90) (Falandysz et al. 2003c; Maćkiewicz and Falandysz 2012). These data on *Tricholoma* mushrooms showed their good ability for absorption of Hg from soil substrate. An open question for *Tricholoma* mushrooms is the difference in availability between the geogenic Hg in lateritic red earths and red and yellow earths and the anthropogenic Hg deposited in the upper layer of soils.

### Possible implications for consumers

Several species of edible wild-growing mushrooms from Yunnan and Minya Konka in Sichuan in China for which data on contamination with Hg were available for the first time showed elevated contents of this element in the fruiting bodies, which are one to two orders of magnitude greater when compared to staple vegetable foods. Important factors to consider are the culinary processing used in Yunnan to prepare a dish which may impact the fate of the Hg contained in mushrooms and also the bio-availability and bio-accessibility of Hg from a meal made of mushrooms high in Hg as well as the role of Se in mushrooms naturally rich in Hg. Consumption of mushrooms foraged by people of Yunnan is one of the highest in the world and in some locations exceeds 20 kg per capita annually (Zhang et al. 2010). Yunnan province is exceptionally rich in species of macrofungi and is called “the kingdom of mushrooms”. Mushrooms are commonly foraged everywhere across Yunnan and reach local regional and distant markets in China and abroad (especially in the remote region of the Shangri-la in the north) (Wang et al. 2014). If based on standard measures of Hg intake and risk such as the value of the provisionally tolerable weekly intake (PTWI) or the reference dose (RfD), the many edible mushrooms from Yunnan provide a high dose of Hg when consumed at a rate of 300 g or more per week.

## Conclusions

Mercury content in the red and yellow lateritic soils in the forested regions of Yunnan Province in southwestern China is elevated over the background level due to their location in the Circum-Pacific Global Mercuriferous Belt. Mercury content in forest soil from the subalpine section of the Minya Konka summit in the Eastern Tibetan Plateau is considered to be elevated over the background levels because of the susceptibility of the region to deposition of airborne Hg originating from distant sources. A large majority of the fungal species examined have no previous data on accumulation of Hg in fruiting bodies from either China, Asia or elsewhere.

The BCF values for Hg for most mushrooms from Yunnan, for which there were available data on Hg in soil, showed on bio-inclusion and values up to 26 (for caps) and 16 (for stipes) for *C. xanthophylla.* Most of the mushrooms in this study with available data on Hg in their soil substrate bio-included Hg. Geogenic Hg from the mercuriferous belt seems to be the overriding source of mercury accumulated in mushrooms foraged in the regions of Yunnan, while long-range atmospheric transport and subsequent deposition are the mercury sources for specimens foraged in the region of Minya Konka.

## References

[CR1] Árvay J, Tomáša J, Hauptvogl M, Kopernická M, Kováčik A, Bajčan D, Massányi P (2014). Contamination of wild-grown edible mushrooms by heavy metals in a former mercury-mining area. J Environ Sci Health Part B.

[CR2] Bargagli R, Baldi F (1984) Mercury and methyl mercury in higher fungi and their relation with the substrata in a cinnabar mining area. Chemosphere 13(9):1059–1071

[CR3] Brzostowski A, Jarzyńska G, Kojta AK, Wydmańska D, Falandysz J (2011). Variations in metal levels accumulated in Poison Pax (*Paxillus involutus*) mushroom collected at one site over four years. J Environ Sci Health Part A.

[CR4] Cenci RM, Cocchi L, Petrini O, Sena F, Siniscalco C, Vescovi L (2009) Elementi chimici nei funghi superiori I funghi di riferimento come strumento di lavoro per la bioindicazione e la biodiversità. JRC Sci Tech Rep EUR 24415 IT 2009

[CR5] Chudzyński K, Bielawski L, Falandysz J (2009). Mercury bio-concentration potential of larch bolete, *Suillus grevillei*, mushroom. Bull Environ Contam Toxicol.

[CR6] Chudzyński K, Jarzyńska G, Stefańska A, Falandysz J (2011). Mercury content and bio-concentration potential of Slippery Jack, *Suillus luteus*, mushroom. Food Chem.

[CR7] Drewnowska M, Jarzyńska G, Kojta AK, Falandysz J (2012). Mercury in European Blusher, *Amanita rubescens*, mushroom and soil. Bioconcentration potential and intake assessment. J Environ Sci Health Part B.

[CR8] Drewnowska M, Lipka K, Jarzyńska G, Danisiewicz-Czupryńska D, Falandysz J (2013) Investigation on metallic elements in fungus *Amanita muscaria* (fly agaric) and the forest soils from the Mazurian lakes district of Poland. Fresenius Environ Bull 22:455–460

[CR9] Drewnowska M, Nnorom IC, Falandysz J (2014). Mercury in the Tawny Grisette, *Amanita vaginata* Fr. and soil below the fruiting bodies. J Environ Sci Health Part B.

[CR10] Falandysz J (2016). Distribution of mercury in Gypsy *Cortinarius caperatus* mushrooms from several populations: an efficient accumulator species and estimated intake of element. Ecotox Environ Saf.

[CR11] Falandysz J, Bielawski L (2007). Mercury and its bioconcentration factors in Brown Birch Scaber Stalk (*Leccinum scabrum*) from various sites in Poland. Food Chem.

[CR12] Falandysz J, Brzostowski A (2007). Mercury and its bioconcentration factors in Poison Pax (*Paxillus involutus*) from various sites in Poland. J Environ Sci Health Part A.

[CR13] Falandysz J, Chwir A (1997). The concentrations and bioconcentration factors of mercury in mushrooms from the Mierzeja Wiślana sand-bar, northern Poland. Sci Total Environ.

[CR14] Falandysz J, Drewnowska M (2015). Distribution of mercury in *Amanita fulva* (Schaeff.) Secr. mushrooms: accumulation, loss in cooking and dietary intake. Ecotox Environ Saf.

[CR15] Falandysz J, Gucia M, Frankowska A, Kawano M, Skwarzec B (2001). Total mercury in wild mushrooms and underlying soil substrate from the city of Umeå and its surroundings, Sweden. Bull Environ Contam Toxicol.

[CR16] Falandysz J, Szymczyk K, Ichihashi H, Bielawski L, Gucia M, Frankowska A, Yamasaki S (2001). ICP/MS and ICP/AES elemental analysis (38 elements) of edible wild mushrooms growing in Poland. Food Addit Contam.

[CR17] Falandysz J, Lipka K, Kawano M, Brzostowski A, Dadej M, Jędrusiak A, Puzyn T (2003). Mercury content and its bioconcentration factors at Łukta and Morąg, northeastern Poland. J Agric Food Chem.

[CR18] Falandysz J, Gucia M, Brzostowski A, Kawano M, Bielawski L, Frankowska A, Wyrzykowska B (2003). Content and bioconcentration of mercury in mushrooms from northern Poland. Food Addit Contam.

[CR19] Falandysz J, Brzostowski A, Kawano M, Kannan K, Puzyn T, Lipka K (2003). Concentrations of mercury in wild growing higher fungi and underlying substrate near Lake Wdzydze, Poland. Water Air Soil Pollution.

[CR20] Falandysz J, Kunito T, Kubota R, Bielawski L, Mazur A, Falandysz JJ, Tanabe S (2007). Selected elements in Brown Birch Scaber Stalk *Leccinum scabrum*. J Environ Sci Health Part A.

[CR21] Falandysz J, Dryżałowska A, Saba M, Wang J, Zhang D (2014). Mercury in the fairy-ring of *Gymnopus erythropus* (Pers.) and *Marasmius dryophilus* (Bull.) P. Karst. mushrooms from the Gongga Mountain, Eastern Tibetan Plateau. Ecotoxicol Environm Saf.

[CR22] Falandysz J, Zhang J, Wang Y, Krasińska G, Kojta A, Saba M, Shen T, Li T, Liu H (2015). Evaluation of the mercury contamination in mushrooms of genus *Leccinum* from two different regions of the world: accumulation, distribution and probable dietary intake. Sci Total Environ.

[CR23] Falandysz J, Zhang J, Wang Y, Saba M, Krasińska G, Wiejak A, Shen T, Li T (2015). Evaluation of mercury contamination in fungi boletus species from latosols, lateritic red earths, and red and yellow earths in the Circum-Pacific Mercuriferous Belt of southwestern China. PLoS One.

[CR24] Fan C (1991). A study on the origin of ore-forming materials in the antimony and mercury multiple-metal ore zone at Weisha, Yunnan Province, China (*in Chinese*). Geochemica.

[CR25] Fischer RG, Rapsomanikis S, Andreae MO, Baldi F (1995). Bioaccumulation of methylmercury and transformation of inorganic mercury by macrofungi. Environ Sci Technol.

[CR26] Fu X, Feng X, Zhu W, Wang S, Lu J (2008). Total gaseous mercury concentrations in ambient air in the eastern slope of Mt. Gongga, south-eastern fringe of the Tibetan plateau, China. Atmosph Environ.

[CR27] Gucia M, Jarzyńska G, Kojta AK, Falandysz J (2012). Temporal variability in twenty chemical elements content of Parasol Mushroom (*Macrolepiota procera*) collected from two sites over a few years. J Environ Sci Health Part B.

[CR28] Gustin M, Lindberg S, Marsik F, Casimir A, Ebinghaus R, Edwards G, Hubble-Fitzgerald C, Kemp R, Kock H, Leonard T, London J, Majewski M, Montecinos C, Owens J, Pilote M, Poissant L, Rasmussen P, Schaedlich F, Schneeberger D, Schroeder W, Sommar J, Turner R, Vette A, Wallschlaeger D, Xiao Z, Zhang H (1999). Nevada STORMS project: measurement of mercury emissions from naturally enriched surfaces. J Geophys Res.

[CR29] He Z, Zhang M, Wilson MJ, Wilson MJ (2004). Distribution and classification of red soils in China. The red soils of China.

[CR30] Index Fungorum (2015) http://www.indexfungorum.org/Names/Names.asp; retrieved on Feb. 30, 2015

[CR31] Kalač P, Nižnanská M, Bevilaqua D, Stašková I (1996). Concentration of mercury, copper, cadmium and lead in fruiting bodies of edible mushrooms in the vicinity of a mercury smelter and a copper smelter. Sci Total Environ.

[CR32] Kojta AK, Jarzyńska G, Falandysz J (2012) Mineral composition and heavy metal accumulation capacity of Bay Bolete (*Xerocomus badius*) fruiting bodies collected near a former gold and copper mining area. J Geochem Explor 121:76–82

[CR33] Kojta AK, Zhang J, Wang Y, Li T, Saba M, Falandysz J (2015). Mercury contamination of fungi genus *Xerocomus* in the Yunnan Province in China and the region of Europe. J Environ Sci Health Part A.

[CR34] Maćkiewicz D, Falandysz (2012). Total mercury in Yellow Knights (*Tricholoma equestre*) mushrooms and beneath soils. Bull Environ Contam Toxicol.

[CR35] Mao X (2009) Macromycetes of China. China Press, ISBN 10:7030244133/ISBN 13:9787030244130

[CR36] Melgar MJ, Alonso J, García MÁ (2009). Mercury in edible mushrooms and soil. Bioconcentration factors and toxicological risk. Sci Total Environ.

[CR37] Michelot D, Melendez-Howell LM (2003). *Amanita muscaria*, chemistry, biology, toxicology and ethnomycology. Mycol Res Pt.

[CR38] Mleczek M, Siwulski M, Stuper-Szablewska K, Rissmann I, Sobieralski K, Goliński P (2013). Accumulation of elements by edible mushroom species: part I. Problem of trace element toxicity in mushrooms. J Environ Sci Health Part B.

[CR39] Mleczek M, Siwulski M, Mikołajczak P, Gasiecka M, Sobieralski K, Szymańczyk M, Goliński P (2015). Content of selected elements in *Boletus badiu*s fruiting bodies growing in extremely polluted wastes. J Environ Sci Health Part A.

[CR40] Nnorom IC, Jarzyńska G, Drewnowska M, Kojta AK, Pankavec S, Falandysz J (2013). Trace elements in sclerotium of *Pleurotus tuber-regium* (Ósu) mushroom—dietary intake and risk in southeastern Nigeria. J Food Compos Anal.

[CR41] Olivero-Verbel J, Johnson B, Arguello E (2002). Human exposure to mercury in san Jorge river basin, Colombia (South America). Sci Total Environ.

[CR42] Olivero-Verbel J, Cabarello-Gallardo K, Turizo-Tapia A (2015). Mercury in the gold mining district of San Martin de Loba, South of Bolivar (Colombia). Environ Sci Poll Res.

[CR43] Ostos C, Perez-Rodriguez F, Moreno Arroyo B, Moreno-Rojas R (2015). Study of mercury content in wild edible mushrooms and its contribution to the provisional tolerable weekly intake in Spain. J Food Comp Anal.

[CR44] Qiu G, Feng X, Wenig B, Sommar J, Gu C (2012). Environmental geochemistry of an active Hg mine in Xunyang, Shaanxi Province, China. Appl Geochem.

[CR45] Rytuba JJ (2003). Mercury from mineral deposits and potential environmental impact. Environ Geol.

[CR46] Saba M, Falandysz J, Nnorom IC (2016). Accumulation and distribution of mercury in fruiting bodies by fungus *Suillus luteus* foraged in Poland, Belarus and Sweden. Environ Sci Poll Res.

[CR47] Saba M, Falandysz J, Nnorom IC (2016b) Evaluation of vulnerability of Suillus variegatus and Suillus granulatus mushrooms’ to sequester mercury in fruiting bodies. J Environ Sci Health Part B 51:540–545 10.1080/03601234.2016.1170552 27166831

[CR48] Saba M, Falandysz J, Nnorom IC (2016c) Mercury determination in *Suillus bovinus* mushroom: accumulation, distribution, probable dietary intake. Environ Sci Poll Res 23:14549–14559 10.1007/s11356-016-6558-8 PMC4943988 27068912

[CR49] Seeger R, Nützel R (1976). Quecksilbergehalt der Pilze. Zeitschr Lebensm Untersuch Forsch.

[CR50] Shi J, Meng M, Shao M, Zhang Z, Zhang Q, Jiang G (2013). Spatial distribution of mercury in topsoil from five regions of China. Environ Sci Poll Res.

[CR51] Suchara I, Sucharová J (2002). Distribution of sulphur and heavy metals in forest floor humus of the Czech Republic. Water Air Soil Pollution.

[CR52] Sun H, Wu Y, Yu D, Zhou J (2013). Altitudinal gradient of microbial biomass phosphorus and its relationship with microbial biomass carbon, nitrogen, and rhizosphere soil phosphorus on the eastern slope of Gongga mountain, SW China. PLoS One.

[CR53] Svoboda L, Havličkova B, Kalač P (2006). Contents of cadmium, mercury and lead in edible mushrooms growing in a historical silver-mining area. Food Chem.

[CR54] Tipping E, Lofts S, Hooper H, Frey B, Spurgeon D, Svendsen C (2010). Critical limits for Hg (II) in soils, derived from chronic toxicity data. Environ Pollut.

[CR55] Tüzen M, Soylak M (2005). Mercury contamination in mushroom samples from Tokat, Turkey. Bull Environ Contam Toxicol.

[CR56] Vetter J, Berta E (1997). Mercury content of some wild edible mushrooms. Zeitschr-Lebensm Untersuch-Forschung.

[CR57] Wang Y, Zhang J, Wu L, Zhao Y, Li T, Li J, Wang Y, Liu H (2014). A mini-review of chemical composition and nutritional value of edible wild-grown mushroom from China. Food Chem.

[CR58] Wen X, Chi Q (2007). Geochemical spatial distribution of mercury in China. Geochimica.

[CR59] Wu Y, Li W, Zhou J, Cao Y (2013). Temperature and precipitation at two meteorological stations on eastern slope of Gongga Mountain, SW China in the past two decades. J Mt Sci.

[CR60] Zhang Q, Huang J, Wang F, Xu J, Mark L, Armstrong D, Li C, Zhang Y, Kang S (2008). Mercury distribution and deposition in glacier snow over western China. Environ Sci Technol.

[CR61] Zhang D, Frankowska A, Jarzyńska G, Kojta AK, Drewnowska M, Wydmańska D, Bielawski L, Wang J, Falandysz J (2010) Metals of King Bolete (*Boletus edulis*) collected at the same site over two years. African J Agricult Res 5:3050–3055

[CR62] Zhang H, Yin R, Feng X, Sommar J, Anderson CWN, Sapkota A, Fu X, Larssen T (2013). Atmospheric mercury inputs in montane soils increase with elevation: evidence from mercury isotope signatures. Scientific Reports.

